# The Clinical Frailty Scale is a valid and independent predictor of one-year survival in patients sustaining a hip fracture: scottish Hip Fracture Audit data from 8,092 patients

**DOI:** 10.1302/2633-1462.612.BJO-2025-0198.R1

**Published:** 2025-12-04

**Authors:** Matthew J. Kennedy, Rose S. Penfold, Lorraine Donaldson, Andrew J. Hall, Martin J. Davison, Alasdair M. J. MacLullich, Phil Walmsley, Nick D. Clement, Jon V. Clarke

**Affiliations:** 1 Scottish Hip Fracture Audit, Public Health Scotland, Edinburgh, UK; 2 Golden Jubilee University National Hospital, Clydebank, UK; 3 School of Medicine, University of St Andrews, St Andrews, UK; 4 Ageing and Health, Usher Institute, University of Edinburgh & Advanced Care Research Centre, Usher Institute, University of Edinburgh, Edinburgh, UK; 5 Department of Orthopaedics & Trauma, University Hospital Wishaw, Wishaw, UK; 6 National Treatment Centre, Victoria Hospital, Kirkcaldy, UK; 7 Department of Orthopaedics & Trauma, Royal Infirmary of Edinburgh, Edinburgh, UK; 8 Department of Biomedical Engineering, University of Strathclyde, Glasgow, UK

**Keywords:** Hip Fracture, Frailty, Clinical frailty scale, Hip fracture registry, Scottish hip fracture audit, Hip fractures, Anesthesiologists, frailty, logistic regression analysis, Multivariable Cox regression analysis, clinicians, strengths, retrospective cohort studies, orthopaedic surgeon

## Abstract

**Aims:**

Hip fracture patients have a significant mortality risk. Risk stratification tools are important in guiding management and family discussions. Aims were to assess the associations and validity of the Clinical Frailty Scale (CFS) in predicting mortality and return to original residence within 30 days using national hip fracture registry data.

**Methods:**

Routinely collected clinical registry data for all patients presenting with a hip fracture in Scotland aged 50 years and over between February 2022 and December 2023 with a completed CFS score were analyzed. The association of frailty with mortality and return to original residence was assessed using multivariable Cox regression and logistic regression analysis, respectively, adjusting for confounders to present adjusted hazard (aHRs) and odds ratios (aORs).

**Results:**

Of 15,546 patients, 8,573 had completed the CFS. Exclusion for missingness gave a final sample of 8,092. Most (71.4%) were female with a median American Society of Anesthesiologists (ASA) grade of 3 (IQR 3 to 3) and CFS of 5 (IQR 4 to 7). Vulnerable and frail patients (CFS ≥ 4) were older, more likely to be admitted from a higher care setting, and had increased mortality risk on the same admission. Higher CFS scores were associated with increased mortality risk: mildly frail (CFS 4 to 5), aHR 1.67 (95% CI 1.53 to 1.87); and frail (CFS 6 to 8), aHR 3.01 (95% CI 2.59 to 3.50). CFS and ASA grade showed similar performance in predicting one-year mortality (CFS area under curve (AUC) 0.72, 95% CI 0.71 to 0.73; ASA AUC 0.66, 95% CI 0.65 to 0.67) and return to residence (CFS AUC 0.63, 95% CI 0.62 to 0.65; ASA AUC 0.61, 95% CI 0.60 to 0.62).

**Conclusion:**

The CFS is a pragmatic and validated tool for assessing frailty, which has a strong association with mortality risk in patients with hip fractures. Its predictive accuracy supports its integration into national hip fracture registries. While its utility in predicting return to pre-injury residence is moderate, it remains a valuable component of comprehensive patient assessment.

Cite this article: *Bone Jt Open* 2025;6(12):1550–1558.

## Introduction

Hip fractures are associated with an elderly patient demographic and are associated with increased mortality rates at 30 days and one-year.^1,2^ Individuals sustaining a hip fracture often have pre-existing frailty, dementia, or delirium, or combinations thereof; all of which are associated with poor outcomes.^3^ Understanding mortality risk in individual patients is important to inform discussions with patients and carers. This has led to the development of a number of predictive scores of mortality following hip fracture to guide service planning.^4-7^ The Nottingham Hip Fracture Score (NHFS) is a widely employed example.^7^ However, many of these involve parameters not available on hospital admission, such as biochemical results, and therefore not of practical use in the hip fracture population.

Frailty is a term that broadly refers to a condition of reduced resilience to illness or injury in the context of decreased function and general health, often in association with older age.^8,9^ Several frailty scoring systems have been developed. Many of these are based on retrospective record review and Healthcare claims reviews.^8-12^ Others rely on extensive review and gait analysis.^13^ The Clinical Frailty Scale (CFS)^14^ score is a nine-point scoring system developed to classify a patient based on their function, and use this as a prediction of the multiple impairments which contribute to frailty. The scale is categorical: ‘coping well’ (CFS 1 and 2); ‘vulnerable’ (CFS 3 and 4); ‘mildly frail’ (CFS 4 and 5); ‘moderately frail’ (CFS 6 and 7); ‘severely frail’ (CFS 8), and an additional category for those with ‘terminal illness’ (CFS 9). The scale is based on the patient’s functional status and independence in activities of daily living, and has expanded from Rockwood’s initial scale of seven points to a nine-point scale to recognize patients who are terminally ill but not otherwise frail and not expected to survive longer than six months (CFS 9).^14,15^ Crucially, the score is derived from patient status two weeks before admission, and all parameters for its completion are collected in a detailed clinical or collateral history. It does not require biochemical markers or other laboratory tests for its completion, enabling clinicians to score at the first assessment, which is at odds with many hip fracture predictive scores.^5,7,16^ Its use by non-geriatricians has been validated.^17,18^ Its use has been validated in the care of elderly patients internationally, and its use for surgical patients has become more widely adopted.^9,19,20^ As its use has become more widespread, centres have tested it against labour-intensive hip fracture-specific predictive models and, in the limited cohort studies available, it has been found to be comparable.^21^ In 2022, the Scottish Hip Fracture Audit (SHFA)^22^ became the first national hip fracture registry to include CFS as a data variable for collection, and its utility has not been tested in the Scottish population.

The aim of this study was to assess the associations and validity of the CFS with mortality and return to place of residence in a national hip fracture population.

## Methods

### Study design

This retrospective cohort study used routinely collected clinical registry data linked to national hospital and mortality records. Approval was obtained from NHS National Services Scotland (ID: DP24250172).

### Data sources and participants

The SHFA is a national registry of people hospitalized with an acute hip fracture in Scotland aged 50 years and over. It captures > 99% of the target population. Data were obtained for all patients between the start of 2022 and October 2024. Hip fracture is defined as an extracapsular or intracapsular fracture of the proximal femur, up to and including subtrochanteric (the region extending 5 cm below the lesser trochanter). Periprosthetic, pubic ramus, acetabular, or isolated greater trochanter fractures are excluded from the registry. The start date of 2022 was chosen as the CFS score was added to the SHFA dataset at this time as an observational (non-mandated) variable. Other variables included: demographic details; pre-fracture residence; hospital site; admission date; American Society of Anesthesiologists (ASA) grade;^23^ inpatient care factors including adherence to the SHFA 12 care standards and return to original residence by 30 days;^22^ length of stay; inpatient mortality; and discharge destination.

Missingness was assessed for each routinely collected variable in the dataset. Due to the non-mandatory CFS completion, limited completion was expected. Missingness was assessed and < 3% missingness in each variable was felt to be missing at random (MAR) and unlikely to affect results.

Individual patient-level audit data were linked by a senior Public Health Scotland Data Analyst (LD) through a unique identifier to mortality and Scottish records (SMR01), and by resident postcode to the Scottish Index of Multiple Deprivation (SIMD).^24^

### Participant characteristics

**Fig. 1:**
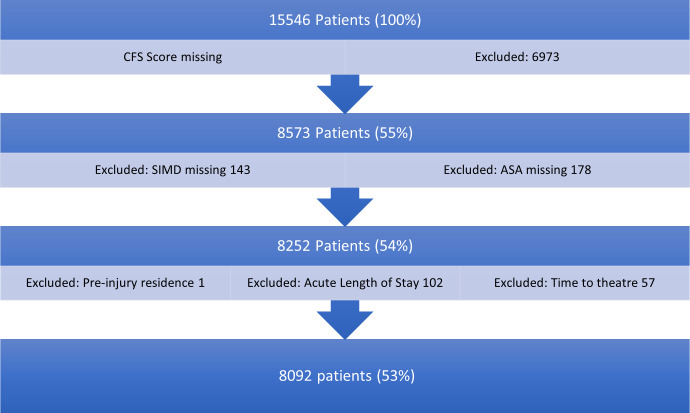
Inclusion flowchart. ASA, American Society of Anesthesiologists; CFS, Clinical Frailty Scale; SIMD, Scottish Index of Multiple Deprivation.

A total of 8,092 patients (mean age 81.7 years (SD 8.8); 71.4% female) presented with a hip fracture, had a CFS score, and reached inclusion criteria regarding missingness (Figure 1). Median CFS score was 5 (IQR 3 to 7) and 6,528 were at least CFS score 4 (‘vulnerable’). Of the 8,573 patients with a completed CFS score, ASA grade was missing for 178 patients (2.1%), SIMD missing for 143 (1.6%), acute length of stay 102 (1.2%), time to theatre 57 (< 0.1%), and pre-injury residence for a single patient (< 0.01%).

Baseline characteristics for the cohort and according to CFS score are shown in Table I .

**Table I.** Baseline characteristics for the cohort and stratified according to Clinical Frailty Scale score.

**Variable**	**Overall, n = 8,092**	**CFS 1, n = 251**	**CFS 2, n = 581**	**CFS 3, n = 1,111**	**CFS 4, n = 1,164**	**CFS 5, n = 1,593**	**CFS 6, n = 1,615**	**CFS 7, n = 1,563**	**CFS 8, n = 170**	**CFS 9, n = 44**	**p-value**
Mean age, yrs (SD)	81.7 (8.8)	74.3 (9.4)	75.5 (8.8)	78.3 (8.2)	80.5 (8.1)	82.6(8.2)	83.7 (8.3)	84.9 (8.0)	85.7 (7.5)	79.5 (8.6)	Df = 1, F value = 1,037, p ≤ 0.001*
Female sex, n (%)	5,775 (71.4)	161 (64.1)	427 (73.5)	806 (72.5)	801 (68.8)	1,129 (70.9)	1,161 (71.9)	1,142 (73.1)	124 (72.9)	24 (54.5)	χ2 = 21.1, Df = 8, p = 0.034
**Pre-fracture residence, n (%)†**											χ2 = 3,117.3, Df = 16, p ≤ 0.001
Own home	6,245 (77.2)	237 (94.4)	561 (96.6)	1,083 (97.5)	1,118 (96.0)	1,474 (92.5)	1,205 (74.6)	495 (31.7)	45 (26.5)	27 (61.4)	
Care home	1,450 (17.9)	7 (2.8)	11 (1.9)	20 (1.8)	22 (1.89)	56 (3.5)	293 (18.1)	919 (58.8)	112 (65.9)	10 (22.7)	
Higher care setting	397(4.9)	7(2.8)	9(1.5)	8(0.7)	24(2.1)	63(4.0)	117(7.2)	149(9.5)	13(7.6)	7(16.0)	
**ASA grade, n (%)‡**											
1 (healthy)	106 (1.3)		29 (5.0)	26 (2.3)	10 (0.9)	10 (0.6)	3 (0.2)	2 (0.1)	1 (0.6)	0 (0.0)	χ2 = 1,532.2, Df = 32, p ≤ 0.001
2	1,870 (23.1)	25 (10.0)	297 (51.1)	482 (43.4)	326 (28.0)	320 (20.1)	195 (12.1)	117 (7.5)	13 (7.6)	1 (2.3)	
3	4,913 (60.7)	119 (47.4)	228 (39.2)	542 (48.8)	729 (62.6)	1,055 (66.2,	1,105 (68.4)	1,038 (66.4)	104 (61.2)	19 (43.2)	
4	1,192 (14.7)	93 (37.1)	26 (4.5)	61 (5.5)	99 (8.5)	207 (13.0)	310 (19.1)	401 (25.7)	51 (30.0)	23 (52.3)	
5 (moribund) or not ever fit for theatre§	11 (0.1)	14 (5.6)	1 (0.2)	0 (0.0)	0 (0.0)	1 (0.1)	2 (0.1)	5 (0.3)	1 (1.0)	1 (2.3)	
**SIMD quintile, n (%)** ¶											
1 (most deprived)	1,581(19.5)	48 (19.1)	104 (17.9)	201 (18.1)	236 (20.3)	328 (20.6)	319 (19.8)	305 (19.5)	31 (18.2)	9 (20.5)	χ2 = 47.3, D *f* = 32, p = 0.199
2	1,636 (20.2)	42 (16.7)	101 (17.4)	228 (20.5)	227 (19.5)	347 (21.8)	361 (22.4)	285 (18.2)	30 (17.6)	15 (34.1)	
3	1,589 (19.6)	50 (19.9)	121 (20.8)	209 (18.8)	223 (19.2)	317 (19.9)	329 (20.4)	299 (19.1)	36 (21.2)	5 (11.4)	
4	1,593 (19.7)	55 (21.9)	131 (22.5)	233 (21.0)	226 (19.4)	314 (19.7)	281 (17.4)	306 (20.0)	38 (22.4)	9 (20.5)	
5 (least)	1,693 (20.9)	56 (22.3)	124 (21.3)	240 (21.6)	252 (21.6)	287 (18.0)	325 (20.1)	368 (23.5)	35 (20.6)	6 (13.6)	
**Median acute length of stay, days (IQR)** **	10 (7 to 16)	8 (6 to 12)	9.0 (6 to 13)	10.0 (7 to 14)	11.0 (8 to 18)	12.0 (8 to 18)	11.0 (7 to 17)	9.0 (6 to 14)	8.0 (6 to 13)	13.5 (7 to 21)	χ2 = 261.1, D *f* = 8, p ≤ 0.001††
**Discharge destination, n (%)**											
Home	3,100 (38.3)	201 (80.1)	450 (77.5)	796 (71.6)	653 (56.1)	574 (36.0)	302 (18.7)	100 (6.4)	12 (7.1)	12 (27.3)	χ2 = 3,946.4, Df = 24, p ≤ 0.001
Care home	1,284 (15.9)	7 (2.8)	8 (1.4)	17 (1.5)	24 (2.1)	56 (3.5)	260 (16.1)	807 (51.6)	98 (57.6)	7 (15.9)	
Higher care setting	3,410 (42.1)	36 (14.3)	117 (20.1)	281 (25.3)	462 (40.0)	921 (57.8)	970 (60.1)	569 (36.4)	38 (22.4)	16 (36.4)	
Died in hospital	298 (3.7)	7 (2.8)	6 (1.0)	17 (1.5)	25 (2.1)	42 (2.6)	83 (5.1)	87 (5.6)	22 (12.9)	9 (20.5)	

p-values are for differences between groups assessed using chi-squared test, unless otherwise specified, with Bonferroni correction for multiple testing. Higher care settings include inpatient transfers, other acute hospital, or rehabilitation settings.

* Analysis of variance (ANOVA).

† One missing.

‡ 178 missing.

§ Includes both ASA grade 5 and additional Scottish Hip Fracture Audit category ‘not ever fit for theatre’, which may include some delayed presentations and patients not suitable for surgery.

¶ 143 missing.

** 102 missing.

†† Kruskal-Wallis.

ASA, American Society of Anesthesiologists; CFS, Clinical Frailty Scale; df, degrees of freedom; SIMD, Scottish Index of Multiple Deprivation.

### Frailty assessment on admission

The nine-point CFS score for frailty was assessed and attributed to patients on admission as part of routine clinical care. The highest frailty score assigned during the admission was taken as the score used in this study. The nine-point scoring system was grouped to allow multivariable analysis based on trends identified following unadjusted Kaplan-Meier analysis of each CFS score. The groupings were: ‘coping well’ (CFS 1 to 3); ‘vulnerable’ or ‘mildly frail’ (CFS 4 to 5); ‘frail’ (CFS 6 to 8); and ‘terminal illness’ (CFS 9).

### Outcomes

Outcomes were mortality at 30 days and one year, and return to pre-injury residence within 30 days.

### Statistical analysis

Continuous data were compared by analysis of variance (ANOVA) or Kruskal-Wallis tests, categorical by chi-squared tests, and differences between CFS groups by paired comparisons tests with Bonferroni adjustment for multiple testing. Unadjusted Kaplan-Meier survival analysis was performed to estimate survival, stratified by CFS score group. Cox regression survival analysis was performed with CFS 1 to 3 as the reference group to assess the association between CFS and mortality, adjusting for age, sex, pre-fracture residence (home, care home, higher care setting), ASA grade, and SIMD quintile. These same confounders were used in logistic regression analysis for return to original residence within 30 days.

Statistical analysis were performed using R v. 4.3.1 (R Foundation for Statistical Computing, Austria) and packages survminer, survival, tidyverse and stats.

Receiver outcome characteristic (ROC) analysis was performed, comparing CFS score and ASA grade, with area under the curve (AUC) being calculated for each respectively, to assess discrimination prediction. Statistical significance was set at p < 0.05.

## Results

All characteristics differed significantly between groups (Table I). Compared with those who were ‘coping well’ (CFS 1 to 3), patients sustaining a hip fracture and frailty were more likely to be female and older. There was a non-linear relationship between increasing frailty and length of stay. However, median length of stay (LOS) increased until patients were ‘mildly frail’ (CFS 5, acute LOS 12.0 (IQR 8 to 18)) and then trended downwards (CFS 8, acute LOS 8.0 (IQR 6 to 13)).

### Mortality

**Fig. 2:**
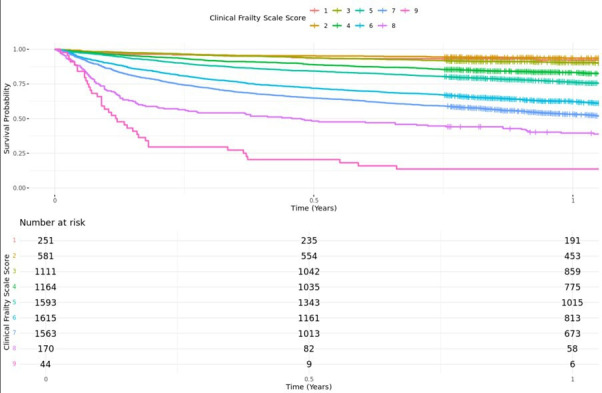
Unadjusted Kaplan-Meier analysis stratified by Clinical Frailty Scale score.

**Fig. 3:**
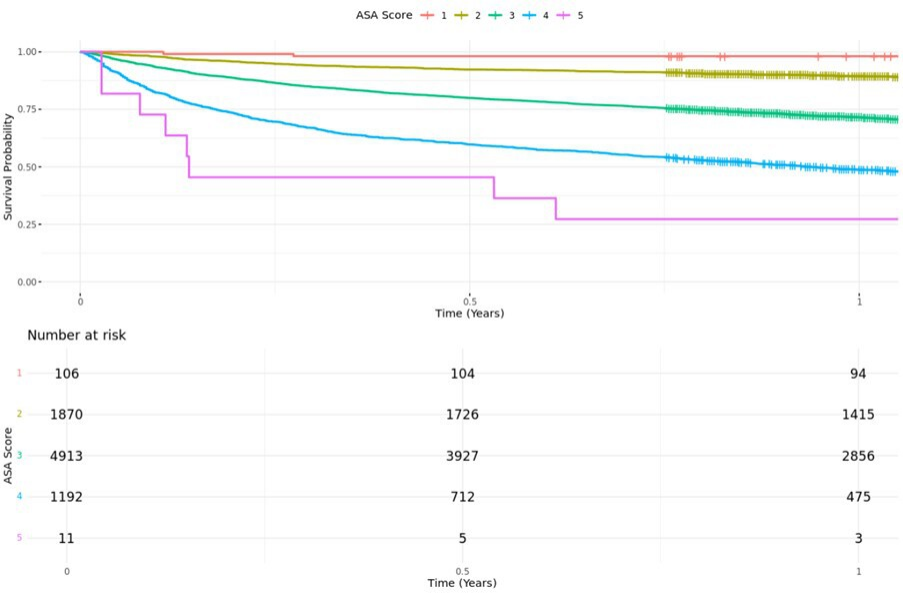
Unadjusted Kaplan-Meier analysis stratified by American Association of Anesthesiologists (ASA) grade.

Unadjusted Kaplan-Meier survival curves (Figure 2 and Figure 3) demonstrated lower survival rates among patients with higher frailty scores and ASA grade, with the greatest difference in the initial period following hip fracture. Multivariable Cox regression analysis, adjusting for age, ASA grade, sex, deprivation, and pre-hospital residence, demonstrated that increasing CFS score was associated with increased mortality (Table II). Patients who were ‘vulnerable’ or ‘mildly frail’ (CFS4 to 5) had almost double the mortality risk (aHR 1.77, 95% CI 1.52 to 2.06) when compared with their ‘coping well’ counterparts (CFS 1 to 3). Similarly, moderate/severe frailty was associated with a threefold mortality risk (2.59 to 3.50), and ‘terminal illness’ (CFS 9) associated with nearly ten-fold (aHR 9.95, 95% CI 7.03 to 14.09) mortality risk compared with the ‘coping well’ group (CFS 1 to 3), respectively.

**Table II.** Cox regression analysis of mortality at one-year and multi-level logistic regression analysis of return to original place of residence within 30 days.

	Cox regression analysis, mortality			Multi-level logistic regression analysis, return to original place of residence within 30 days		
Variable	Unadjusted HR (95% CI)	Adjusted HR (95% CI)	p-value	Unadjusted OR (95% CI)	Adjusted OR (95% CI)	p-value
Age, yrs	1.06 (1.05 to 1.06)	1.04 (1.03 to 1.04)	< 0.001	0.95 (0.94 to 0.95)	0.95 (0.94 to 0.96)	< 0.001
**ASA grade**						
1	0.08 (0.03 to 0.22)	0.18 (0.08 to 0.54)	< 0.001	5.71 (3.31 to 10.73)	2.89 (1.61 to 5.62)	< 0.001
2	0.37 (0.33 to 0.42)	0.61 (0.53 to 0.69)	< 0.001	2.52 (2.23 to 2.84)	1.95 (1.70 to 2.23)	< 0.001
3	Ref	Ref	Ref	Ref	Ref	Ref
4	2.10 (1.93 to 2.30)	1.67 (1.53 to 1.83)	< 0.001	0.65 (0.57 to 0.74)	0.65 (0.56 to 0.76)	< 0.001
5*	4.68 (2.52 to 8.72)	2.92 (1.57 to 5.49)	< 0.001	0.80 (0.19 to 3.38)	0.83 (0.16 to 4.24)	0.817
**CFS**						
1 to 3	Ref	Ref	Ref	Ref	Ref	Rer
4 to 5	2.53 (2.18 to 2.94)	1.77 (1.52 to 2.06)	< 0.001	0.28 (0.24 to 0.32)	0.39 (0.34 to 0.46)	< 0.001
6 to 8	5.94 (5.17 to 6.82)	3.01 (2.59 to 3.50)	< 0.001	0.21 (0.18 to 0.24)	0.15 (0.12 to 0.17)	< 0.001
9	21.75 (15.47 to 30.58)	9.95 (7.03 to 14.09)	< 0.001	0.15 (0.08 to 0.29)	0.14 (0.06 to 0.31)	< 0.001
**Sex**						
Female	Ref	Ref	Ref	Ref	Ref	Ref
Male	1.46 (1.35 to 1.58)	1.60 (1.48 to 1.74)	< 0.001	0.79 (0.72 to 0.88)	0.73 (0.65 to 0.82)	< 0.001
**SIMD**						
1 (most deprived)	Ref	Ref	Ref	Ref	Ref	Ref
2	1.00 (0.88 to 1.12)	0.99 (0.88 to 1.12)	0.928	0.85 (0.73 to 0.98)	0.90 (0.76 to 1.06)	0.198
3	1.00 (0.89 to 1.13)	0.97 (0.86 to 1.09)	0.567	0.95 (0.82 to 1.10)	0.92 (0.78 to 1.09)	0.347
4	0.92 (0.82 to 1.04)	0.92 (0.81 to 1.04)	0.161	1.06 (0.92 to 1.23)	1.02 (0.86 to 1.20)	0.827
5 (least deprived)	0.95 (0.84 to 1.07)	0.86 (0.76 to 0.97)	0.013	0.95 (0.82 to 1.10)	0.92 (0.78 to 1.08)	0.305
**Pre-injury residence**						
Home	Ref	Ref	Ref	Ref	Ref	Ref
Care home	2.72 (2.50 to 2.96)	1.38 (1.25 to 1.52)	< 0.001	2.58 (2.27 to 2.95)	10.07 (8.54 to 11.90)	< 0.001
Higher care setting	2.85 (2.48 to 3.27)	1.67 (1.44 to 1.92)	< 0.001	N/A†	N/A†	

* ASA 5 or greater; Scottish Hip Fracture Audit also includes ASA 6 ‘never fit for surgery’.

† 397 admitted from higher care setting were excluded from logistic regression of return to original place of residence analysis.

ASA, American Society of Anesthesiologists; CFS, Clinical Frailty Scale; HR, hazard ratio; N/A, not applicable; OR, odds ratio; SIMD, Scottish Index of Multiple Deprivation.

Patients admitted from care homes (aHR 1.38, 95% CI 1.25 to 1.52) or a higher care setting (aHR 1.67, 95% CI 1.44 to 1.92) both had higher mortality risks than those admitted from home. Those in the least deprived SIMD quintile, had significantly reduced mortality risk (aHR 0.86 95% CI 0.76 to 0.97, p-value 0.013) compared with the most deprived. Otherwise, there was no significant difference between SIMD quintiles.

### Predictive discrimination of mortality

**Fig. 4:**
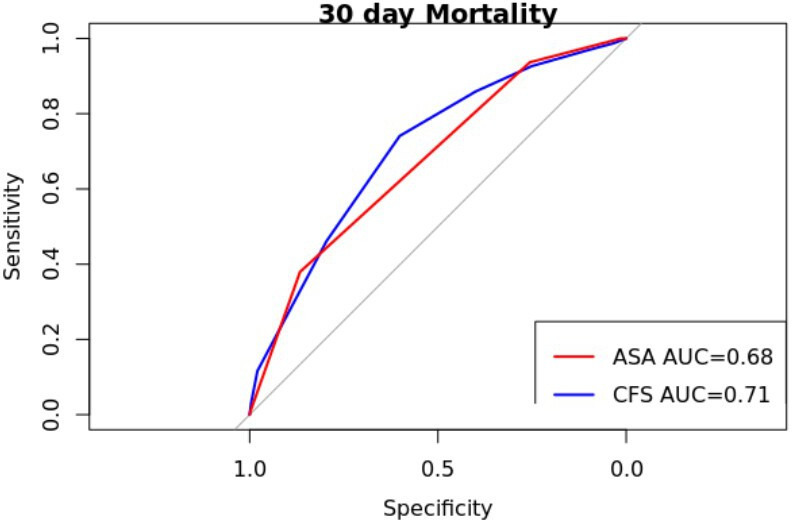
Receiver operator characteristic for 30-day mortality with comparison of Clinical Frailty Scale (CFS) and American Association of Anesthesiologists (ASA) scores, respectively.

**Fig. 5:**
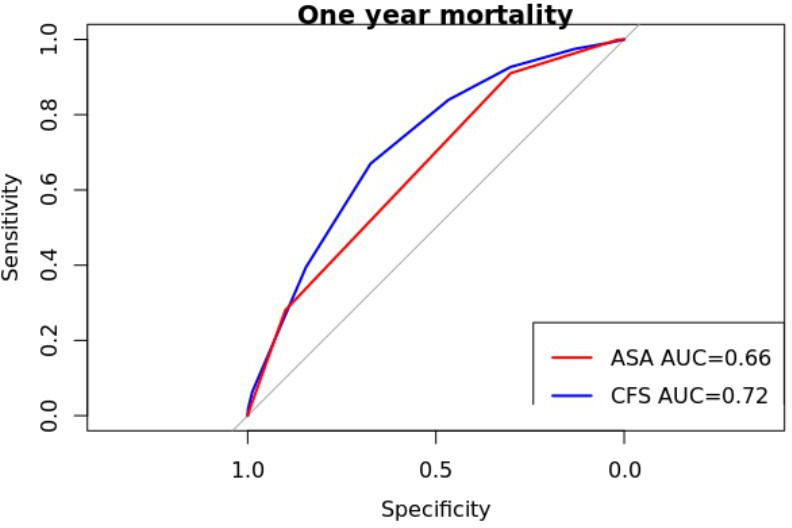
Receiver operator characteristic for one-year mortality with comparison of Clinical Frailty Scale (CFS) and American Association of Anesthesiologists (ASA) scores.

Receiver operator characteristics (ROC) analysis and subsequent area under the curve (AUC) are shown in Figure 4 and Figure 5. CFS score had marginally better validity as a predictor of both 30-day and one-year mortality (30 days AUC 0.71, 95% CI 0.68 to 0.73; one year AUC 0.72, 95% CI 0.71 to 0.73) than ASA (30 days AUC 0.68, 95% CI 0.65 to 0.73; one year AUC 0.66, 95% CI 0.65 to 0.67).

### Return to original residence within 30 days

Multivariable logistic regression analysis demonstrated that increasing age (aOR 0.95, 95% CI 0.94 to 0.96), male sex (aOR 0.73, 95% CI 0.65 to 0.82), ASA 4 (aOR 0.65, 95% CI 0.56 to 0.76), and frailty (CFS 4 to 5 aOR 0.39, 95% CI 0.34 to 0.46; CFS 6 to 8, 95% CI 0.12 to 0.17; CFS 9 95% CI 0.06 to 0.31) were independently associated with not returning to original residence within 30 days (Table II).

The same analysis showed a ten-times increased return to original residence within 30 days in those admitted from a care home compared with home dwellers (aOR 10.07, 95% CI 8.54 to 11.90). There was no predictive value of deprivation on return to original residence.

### Predictive discrimination of return to original residence within 30 days

**Fig. 6:**
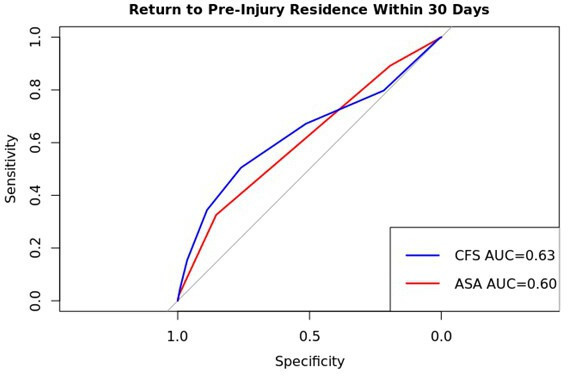
Receiver operator characteristic analysis of return to original residence within 30 days with comparison of Clinical Frailty Scale (CFS) and American Association of Anesthesiologists (ASA) scores.

ROC analysis and subsequent AUC are shown in Figure 6. CFS score (AUC 0.63, 95% CI 0.62 to 0.65) was a better predictive discriminator of return to original residence within 30 days than ASA (AUC 0.61, 95% CI 0.60 to 0.62).

## Discussion

The majority of patients (6,149, 76%) sustaining a hip fracture in Scotland were ‘vulnerable’ (CFS 4) or ‘frail’ (CFS ≤ 5) during the study period. Level of clinical frailty was independently associated with mortality risk and return to original residence within 30 days in patients sustaining a hip fracture, and was the most reliable discriminator of mortality.

### Mortality

This study is the largest reported use of the CFS as an independent predictor of mortality and return to original residence in hip fracture patients. CFS is a reproducible and reliable scoring system.^15,21,25,26^ We found that as frailty increased, the risk of mortality increased. Those who were ‘vulnerable’ or ‘mildly frail’ (CFS 4 to 5) had double and those moderate to ‘severely frail’ (CFS 6 to 8) had three times the increased mortality when compared with the ‘coping well’ (CFS 1 to 3) group. These findings align with a registry-based study of the Australian and New Zealand Hip Fracture Registry (ANZHFR), which found that one-year mortality increased from 3.8% in CFS 1 patients to 41.7% in CFS 7 or greater.^27^ A similar progressive relationship of increasing CFS scores to mortality was found in a single-centre retrospective cohort study of 800 patients with fragility fractures, finding a 55% increase with each increment of CFS for 30-day and one-year mortality.^26^ This differs from the findings of Ikram et al^21^ in a cohort study of 1,255 patients, who found that there was a linear relationship in increasing CFS score 1 to 5, but once the patient was classified as ‘frail’ (CFS 5), there was no discernible increase in mortality, though they were at higher risk of increased length of stay and complications. The available literature suggests that frailty is associated with increased risk of mortality, even if there is no noted increase in mortality with higher CFS scores within the ‘frail’ scoring range. While this study demonstrates increased mortality in patients with higher CFS scores, the results should not be interpreted as an argument for conservative management in these patients, or an argument for mandatory critical care input. Surgical management of hip fractures is well established in making these patients comfortable and enabling them to retain dignity, even in the context of high rates of mortality within 30 days.

### Length of stay and return to pre-injury residence within 30 days

Return to pre-injury residence within 30 days is a key performance indicator in the SHFA Scottish Standard of Care for Hip Fracture Patients (SSCHFP), and is important to patients and carers, alongside mortality.^22^ Length of inpatient stay has been shown not to be correlated with increased mortality and should be analyzed separately.^28^ The finding of increasing acute length of stay with increasing CFS score until ‘mildly frail’ (CFS 5) or ‘moderately frail’ (CFS 6) is in keeping with Ikram et al’s work.^21^ They concluded that the potential challenges of differentiating between mild, moderate, and severe frailty may account for this, but it may also be that these patients have established support networks in place pre-injury and therefore need to wait longer for discharge planning in a publicly funded health service. This interpretation is supported in the multivariable logistic regression analysis results, with pre-injury care home residence being an independent predictor of return to the pre-injury residence. These findings demonstrate that those patients who are vulnerable (CFS 3 to 4) and mildly frail (CFS 5) have a greater demand for comprehensive orthogeriatric input to help decrease their length of stay.

### Comparison of CFS to ASA

The comparative predictors of mortality and return to pre-injury residence was ASA grade. The use of bespoke, research driven, hip fracture prognostic tools (such as the NHFS) is similar to CFS at predicting mortality in two retrospective cohort studies with a combined total of greater than 3,000 patients. AUC analyses of CFS have never approached excellent reliability (AUC ≥ 0.80), with CFS consistently having an AUC of < 0.70.^21,29^ The AUC analysis demonstrated slightly improved discrimination in the prediction of mortality at one year of CFS score compared with ASA (CFS AUC 0.72 (95% CI 0.71 to 0.73), ASA AUC 0.66 (95% CI 0.65 to 0.67)) which is similar to the AUC for NHFS in the literature (AUC 0.69 to 0.7).^29^ This finding is in keeping with Naru et al.^27^ The AUC in this study’s large patient group (AUC > 0.70) suggests that the CFS is the most reliable discriminator in the available literature. A caveat is that all studies conducting similar analyses report comparable discriminative value (AUC > 0.63); therefore, the difference in predictive discrimination is likely small. With regards to return to pre-injury residence within 30 days, there was a difference in the discrimination predictive value of CFS and ASA (CFS AUC 0.63 (95% CI 0.62 to 0.65) (ASA AUC 0.61 (95% CI 0.60 to 0.62)). We believe the important finding is that CFS offers the best prognostic tool available to clinicians currently collected by the audit.

Key strengths of this study include the use of a large national dataset: this enabled a robust sample size (n = 8,092) from a national registry, which enhances generalizability. The ability to link routinely collected audit data to Public Health Scotland (PHS) morbidity and mortality data (SMR01) is also a strength. CFS is widely used tool in multiple settings.^30-32^ This study used appropriate methods, with comprehensive and well-executed statistical analyses (multivariable Cox and logistic regression alongside AUC comparisons). Another strength is the clinically relevant findings reported, with actionable associations between CFS and both mortality and return to residence. A further strength is the comparative analysis with ASA grade which enhances the practical implications of adopting CFS in routine use.

There are limitations to this study. The study relied on routinely collected audit data. The completion of the CFS was 55%. The reason for this is likely due to the recent introduction of this and the lack of incorporating it as a mandatory component of the SSCHFP.^22^ There is no national standard for which the clinician is responsible for CFS scoring being entered into the registry and this may differ between units, with a mix of orthogeriatricians, emergency department, and orthopaedic staff contributing to its completion. Training in CFS application also differed between units with no centralized training model in its use at present. Through the results presented here, we believe this can strengthen the argument to make it part of the agreed national standards for Scottish Hip Fracture Care which would enable national standards in these areas. The retrospective design limits the ability to infer causality. Despite multivariable adjustment, unmeasured variables (e.g. nutritional status, social support) could influence outcomes. While missingness was low (< 3%) for most covariates, non-random missing data cannot entirely be ruled out. The findings presented apply primarily to the Scottish population and may not directly extrapolate to healthcare systems with different structures or patient demographic details. The study lacks functional outcome measures and focused on mortality and return to residence, not assessing important outcomes such as mobility, independence, or quality of life.

In conclusion, three in every four patients presenting with hip fractures are vulnerable or frail. The CFS shows validity as an independent predictor of one-year mortality, performing slightly better than the ASA grade in this large national cohort. It serves as a useful, pragmatic prognostic tool that could be valuable in national hip fracture registries to aid in risk stratification, interpreting outcomes, and guiding clinical discussions. For enhanced risk prediction in clinical practice, future work could explore the use of CFS alongside other established predictors of adverse outcomes that capture different constructs, such as delirium using 4AT. ^33^

**Take home message**

- Frailty is an independent risk factor for increased mortality in hip fracture patients.

- The Clinical Frailty Scale (CFS) score is pragmatic for the orthopaedic surgeon caring for hip fracture patients, with moderate predictive accuracy for 30-day and one-year mortality in a national hip fracture population.

- The CFS should be incorporated into national hip fracture registries.

## Data Availability

The datasets generated and analyzed in the current study are not publicly available due to data protection regulations. Access to data is limited to the researchers who have obtained permission for data processing. Further inquiries can be made to the corresponding author.
